# A novel target capture probe set reveals widespread unknown hybridization in North American pondweeds (Potamogetonaceae)

**DOI:** 10.1002/aps3.70091

**Published:** 2026-11-13

**Authors:** Zacc T. Ramm, Laura A. Frost

**Affiliations:** ^1^ Department of Plant and Microbial Biology University of Minnesota–Twin Cities 1445 Gortner Avenue St. Paul 55108‐1095 Minnesota USA; ^2^ Department of Biology University of South Alabama 5871 USA Drive, North Mobile Alabama 36688‐0002 USA

**Keywords:** hybridization, phylogenomics, *Potamogeton*, Potamogetonaceae, target capture

## Abstract

**Premise:**

Advancements in phylogenomics and bioinformatics provide opportunities to more robustly characterize hybrid lineages and incorporate them into phylogenetic hypotheses. Target capture remains the most cost‐effective molecular method of hybrid determination at scale. The pondweed family is an ecologically important but taxonomically difficult freshwater plant family due to high rates of hybridization. Historically, hybrid pondweeds have been challenging to characterize with limited molecular data, and hybridization is thought to be underdescribed as a result.

**Methods:**

We designed a novel target capture probe set for Potamogetonaceae targeting 350 loci (Pondweeds350), combining 132 custom Potamogetonaceae targets, 205 Angiosperms353 loci, and 13 functional genes for aquatic adaptation. The probe set was tested on 285 samples of North American Potamogetonaceae. HybPhaser was used to identify and phase hybrid accessions for phylogenetic analysis.

**Results:**

Target enrichment was successful for 280 of 285 samples with an average of 336 genes producing contigs per sample. HybPhaser provided evidence for 16 novel hybrid combinations in *Potamogeton*.

**Discussion:**

We demonstrate the effectiveness of the Pondweeds350 probe set as well as the utility of HybPhaser in detecting hybrids at a large scale. We provide evidence for undetected *Potamogeton* hybrids in addition to the most densely sampled and well‐resolved phylogeny of North American Potamogetonaceae to date.

The pondweed family, Potamogetonaceae, is a diverse and taxonomically complex aquatic plant family in the flowering plant order Alismatales (Wiegleb and Kaplan, 1998; Kaplan et al., 2013). Pondweeds have a worldwide distribution, with particularly high species diversity observed in the Northern Hemisphere (Murphy et al., 2019; Fehrer et al., 2022a). As aquatic macrophytes, pondweeds fulfill important roles within their environments such as regulating nutrient cycles, sediment fixation, and serving as both food and habitats for various animal species (Carpenter and Lodge, 1986; Wiegleb et al., 1991). Due to the family's high diversity, wide distribution, and ecological importance, understanding the evolutionary history of Potamogetonaceae should be a priority, but taxonomic confusion has historically hindered these efforts (Wiegleb and Kaplan, 1998; Kaplan and Reveal, 2013; Kaplan et al., 2013; Fehrer et al., 2022a).

Although the number of both genera and species in Potamogetonaceae is debated, the family generally comprises between 80 and 110 species in two to seven genera, depending on whether Zannichelliaceae is included in Potamogetonaceae or not (Haynes et al., 1998; Stevens, 2001; Christenhusz and Byng, 2016; POWO, 2025). The widely agreed upon genera in Potamogetonaceae are *Potamogeton* L., *Stuckenia* Börner, and *Groenlandia* J. Gay (Haynes et al., 1998)—although there is debate about the inclusion of *Stuckenia* in *Potamogeton* (Stevens, 2001)—and the debated genera are *Zannichellia* L., *Althenia* F. Petit, *Pseudalthenia* Nakai (some consider synonymous with *Zannichellia*; POWO, 2025), and *Lepilaena* J. Drumm. ex Harv. (now generally considered *Althenia*; Stevens, 2001; POWO, 2025). We adopt the Plants of the World Online definition of Potamogetonaceae, including *Potamogeton, Stuckenia, Groenlandia, Zannichellia*, and *Althenia* (POWO, 2025).

*Potamogeton* is the largest genus within Potamogetonaceae with approximately 70 currently accepted species (Kaplan and Reveal, 2013; Fehrer et al., 2022b). However, *Potamogeton* remains one of the most difficult aquatic plant groups in which to delimit species due to a combination of phenotypic plasticity, reduced morphology, and broad geographic ranges (Kaplan and Štěpánek, 2003; Kaplan et al., 2013; Fehrer et al., 2022a). Additionally, of all the pondweed genera, *Potamogeton* has an especially well‐documented tendency to hybridize, and there are currently more proposed hybrids (~100 hybrids) than discrete species within the genus (Fehrer et al., 2022b).

Existing molecular studies of Potamogetonaceae have largely focused on identifying and determining the parentage of individual hybrids (Kaplan et al., 2009, 2018; Kaplan and Fehrer, 2011). The studies that have aimed to clarify the diversity of different geographic regions (e.g., Iida et al., 2004; Lindqvist et al., 2006; Wang et al., 2007; Aykurt et al., 2020) have often been limited in taxonomic breadth and sampling of highly polymorphic species. Furthermore, these studies have indicated that even in regions with well‐studied flora, such as North America, there is undetected diversity in Potamogetonaceae (Kaplan et al., 2009, 2018). North America exhibits the highest pondweed diversity, yet much of this diversity is thought to be overlooked or misidentified (Kaplan et al., 2009, 2018; Kaplan and Reveal, 2013).

High levels of phenotypic variation within species can mask intermediate morphologies in hybrids, meaning hybrids are often only reliably identified using morphology by experts in pondweed taxonomy. However, even in these cases, molecular testing is a critical component to identifying putative hybrids (Kaplan et al., 2009, 2011; Kaplan and Fehrer, 2009, 2013). The difficulty with morphological diagnosis also means that hybrids have often only been described where the putative parent species currently overlap. With molecular analysis, hybrid species have been found to occur in environments and geographical areas where one or both parent species are absent, indicating historical hybridization scenarios (Preston et al., 1998; King et al., 2001; Kaplan and Fehrer, 2011; Kaplan et al., 2018). Many of these orphaned hybrids have been overlooked, misidentified, or the parentage misdiagnosed as a result (Kaplan and Fehrer, 2009, 2013; Kaplan et al., 2018). This bias in hybrid detection and toward contemporary hybrid zones likely obscures the true number of hybrids; an in‐depth exploration of *Potamogeton* is warranted to better understand the diversity of the group.

Thus far, molecular methods for hybrid detection and phylogenetic inference of Potamogetonaceae have mainly relied on a small number of Sanger‐sequenced loci, typically the internal transcribed spacer (ITS) region, 5S non‐transcribed spacer (5S‐NTS), and various intergenic chloroplast spacer sequences (Ito et al., 2016; Aykurt et al., 2020; Fehrer et al., 2022a). Previous studies have noted the challenges of finding genetic loci with sufficient variation for robust phylogenetic analysis in Potamogetonaceae (Kaplan and Štěpánek, 2003; Kaplan and Fehrer, 2011; Kaplan et al., 2011). While molecular cloning of the ITS locus can reliably identify hybrids and their parents, this method requires significant wet‐lab time and limits the number of taxa that can be analyzed. Moreover, the drawbacks of relying solely on nuclear spacers for phylogenetic analysis (Álvarez and Wendel, 2003) highlight the need for more robust and reliable molecular tools.

Currently, there are no phylogenetic studies of *Potamogeton* that broadly sample both discrete and hybrid taxa. The phylogenetic studies with the most comprehensive sampling of North American species as well as the best representation across the genus are from Lindqvist et al. (2006) and Ito et al. (2016), but each includes only a single known hybrid sample (*P*. ×*haynesii* Hellq. & G.E. Crow). These studies rely on chloroplast spacer regions and the 5S‐NTS nuclear spacer sequence; the phylogenies they infer are largely poorly resolved. Ito et al. (2016) described the “broad‐leaved” and “linear‐leaved” clades—Clade I and Clade II, respectively, in Lindqvist et al. (2006)—with four subclades each. However, the placement of several North American species remained uncertain and these were not assigned to clades by Ito et al. (Figure 1). These studies provide the current phylogenetic framework for *Potamogeton*, but the monophyly of broad‐leaved and linear‐leaved groups and the relationships among subclades lack support, and placement of several taxa remains unclear.

**Figure 1:**
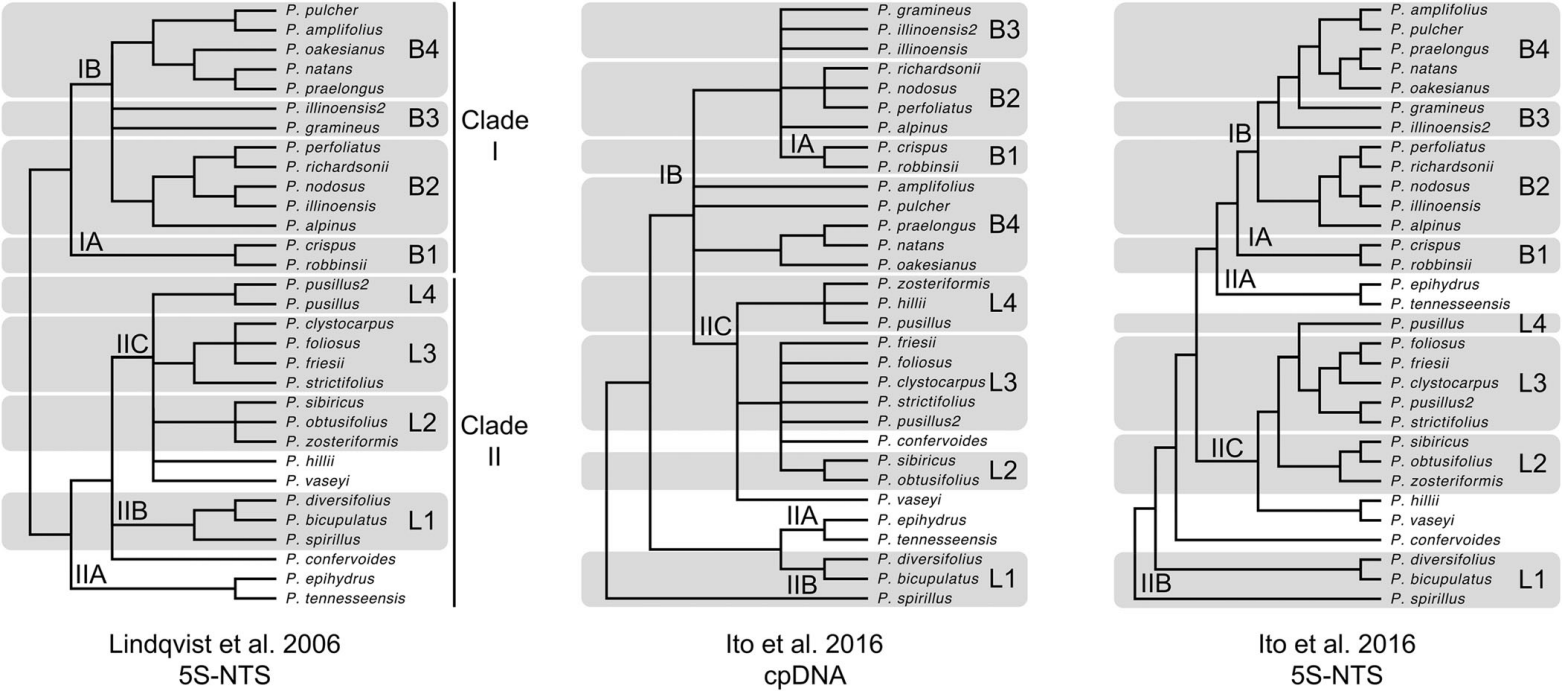
Simplified topologies of previous phylogenetic studies of *Potamogeton* (Lindqvist et al., 2006 [left]; Ito et al., 2016 [center and right]). The study and the genetic markers used to infer the tree are listed below each topology. Subclades are labeled using the naming convention of Lindqvist et al. (2006)—roman numerals and letters—above branches leading to those clades/grades, as well as the naming convention of Ito et al. (2016)—with “B” corresponding to “broad‐leaved” and “L” corresponding to “linear‐leaved” clades and numbers identifying the subclades—in gray boxes highlighting the named clades/grades.

The history of hybridization in Potamogetonaceae is complex, based on the number of described hybrids, the presence of orphaned hybrids, and observed phylogenetic discordance. Genomic tools to resolve relationships within Potamogetonaceae, especially *Potamogeton*, and better detect hybrids will not only improve our systematic understanding of the group, but will also allow exploration of evolutionary questions related to morphology (e.g., the evolution of floating leaves and heterophylly, distinct morphologies between floating and submersed leaves), biogeography, and patterns of hybridization. In light of new phylogenomic tools and hybrid detection methods, we aim to explore the effectiveness of target sequence capture and allele phasing for large‐scale hybrid detection in pondweeds. In this study, we present Pondweeds350, a custom target capture probe set for Potamogetonaceae targeting 350 low‐copy nuclear loci. We generated the first phylogenomic dataset for North American pondweeds (*Potamogeton*, *Stuckenia*, *Zannichellia*), comprising data for 323 nuclear genes for 264 samples that represent >30 discrete taxa as well as numerous hybrids. We utilized the HybPhaser workflow (Nauheimer et al., 2021) to identify and phase hybrid samples for simpler phylogenetic analysis. Our phylogenetic analysis provides the most densely sampled and well‐resolved phylogenetic framework while also highlighting undescribed hybrid combinations.

## METHODS

### Probe design

Custom probes were designed for Potamogetonaceae using the Sondovač pipeline (Schmickl et al., 2016). Sondovač identifies low‐copy nuclear genes using unique transcripts (i.e., those sharing less than 90% sequence similarity) from assembled transcriptome data to extract matching exons from genome skim data with BLAT (Kent, 2002). These BLAT hits are then assembled using Geneious (https://www.geneious.com), and the resulting contigs are filtered for probe sequences of a minimum total target length. Putative targets were identified using the coding sequences from eight Potamogetonaceae transcriptomes generated by Chen et al. (2022) and 13 Potamogetonaceae genome skims downloaded from the National Center for Biotechnology Information (NCBI) Sequence Read Archive (Appendix S1, see Supporting Information with this article). Transcriptomes and genome skims comprised data for species from all three genera of Potamogetonaceae found in North America: *Potamogeton*, *Stuckenia*, and *Zannichellia*. The transcriptomes represented six species of *Potamogeton* (*P. crispus* L., *P. intortusifolius* J.B. He, L.Y. Zhou & H.Q. Wang, *P. maackianus* A. Benn., *P. octandrus* Poir., *P. perfoliatus* L., and *P. wrightii* Morong), *Stuckenia pectinata* (L.) Börner, and *Zannichellia palustris* L. The genome skims represented eight species of *Potamogeton* (*P. alpinus* Balb., *P. berchtoldii* Fieber, *P. friesii* Rupr., *P. gramineus* L., *P. natans* L., *P. obtusifolius* Mert. & W.D.J. Koch, *P. perfoliatus*, and *P. praelongus* Wulfen), two species of *Stuckenia* (*S. filiformis* (Pers.) Börner and *S. vaginata* (Turcz.) Holub), and one accession of *Zannichellia palustris*.

Genome skim reads were pooled based on the established number of chromosomes for each species (Kaplan et al., 2013; Kirina et al., 2025). Potamogetonaceae exhibit a base number (*x*) of either 13 or 14 chromosomes; diploid (2*n* [2*x*] = 26, 28), tetraploid (2*n* [4*x*] = 52), and octaploid (2*n* [8*x*] = 104) species have been observed in *Potamogeton* (Hollingsworth et al., 1998; Kaplan et al., 2013). Despite uncertainty in the phylogenetic relationships of the two major clades of *Potamogeton*, Kaplan et al. (2013) found that species in the linear‐leaved clade are diploid, while most in the broad‐leaved clade are tetraploid, and *Stuckenia* are hexaploid (2*n* [6*x*] = 78). As the polyploid taxa would have different histories of gene duplication and loss, which may affect identification of unique transcripts, we elected to pool them separately. For genome skims of *Potamogeton*, this resulted in two pools: one diploid pool corresponding to the linear‐leaved clade, including *P. berchtoldii*, *P. friesii*, and *P. obtusifolius*, and one tetraploid pool corresponding to the broad‐leaved clade, including reads for *P. alpinus*, *P. gramineus*, *P. natans*, *P. perfoliatus*, and *P. praelongus*. Similarly, the four accessions of *Stuckenia* were pooled together, resulting in four genome skim input files: the diploid/linear‐leaved *Potamogeton* pool, the tetraploid/broad‐leaved *Potamogeton* pool, the hexaploid *Stuckenia* pool, and *Zannichellia palustris* (ploidy varies for this species, but has a base *x* = 12; Kirina et al., 2025). Chloroplast (*P. crispus*, *S. pectinata*, *Z. palustris*) and mitochondrial (*Zostera marina* L., *Butomus umbellatus* L.) genomes were obtained from NCBI GenBank to serve as organellar reference sequences (Appendix S2).

Each of the four genome skim input files was processed against each transcriptome with the *sondovac_part_a.sh* script, resulting in 32 total runs, using the default settings (https://github.com/V-Z/sondovac). Output files from part A of the pipeline were imported into Geneious Prime v2023.2.1 for de novo assembly, and the unique assemblies were processed with the *sondovac_part_b.sh* script. Results from the 32 independent Sondovač runs were combined and highly similar probe sequences were removed using the *cd‐hit‐est* command in CD‐HIT v4.8.1 (Li and Godzik, 2006; Fu et al., 2012) with a 0.9 sequence identity threshold. After combining and removing highly similar targets, probe sequences for 103,655 contigs representing 22,241 transcripts remained. To reduce the number of target sequences and select for genes that may have better performance more broadly in the core Alismatids (as defined by Petersen et al., 2016; Chen et al., 2022), the genome skim input files were run against a *Zostera marina* transcriptome (Appendix S1) from the sister family Zosteraceae. Results of those four independent runs were combined, and highly similar sequences were removed using the *cd‐hit‐est* command.

For some genes, separate targets corresponding to different regions of the same gene were selected in the final output of Sondovač. Because phylogenetic methods assume loci are evolutionarily independent (Jennings, 2017 and citations therein), analyzing multiple targets of the same gene as separate loci violates this assumption. Thus, we aimed to use full coding sequences as target sequences for probe design, which has the benefit of streamlining assembly of multiple target exons—rather than concatenating post‐assembly (Ball et al., 2023)—and preserving exon order. Sondovač targets were locally BLASTed (Basic Local Alignment Search Tool) using the *BLASTn* command in NCBI BLAST+ executables (Camacho et al., 2009) against a combined file with coding sequences from the nine transcriptomes to identify coding sequences corresponding to targets. Because the family has a clear history of genome duplication, as indicated by available chromosome counts (Kaplan et al., 2013), multiple homologous sequences (e.g., alleles, homeologs, or paralogs) were expected even for putatively single‐copy genes. The ‐evalue threshold was set to 1e‐150 to retain multiple, highly similar hits for downstream assessment. This threshold was set based on values observed in initial BLASTn runs without filtering, with the goal that it was neither too strict to obscure multiple gene hits nor so broad as to include non‐homologous genes. Additionally, target sequences from Angiosperms353 were downloaded (https://github.com/mossmatters/Angiosperms353), extracted by gene, and BLASTed to the transcriptomes to add/identify targets that overlap with the widely used, universal angiosperm bait set. Transcripts corresponding to genes also targeted by Angiosperms353 were named following the convention of Angiosperms353, increasing the ability to incorporate data captured by our custom probe set with data generated from studies using the universal bait set. Finally, for a subset of functional genes identified as being related to adaptation to aquatic environments that are present in Potamogetonaceae (Olsen et al., 2016; Han et al., 2019; Chen et al., 2022), *Arabidopsis* protein sequences (Table S6 of Chen et al., 2022) were downloaded and BLASTed to the nine transcriptomes.

For each gene, the full‐length coding sequences for all BLAST hits meeting the ‐evalue 1e‐150 threshold were extracted from the combined file and aligned. Alignments were visualized in AliView v.18.1 (Larsson, 2014) and translated into amino acid (AA) sequences. Alignments were manually adjusted where necessary to preserve the reading frame. At this stage, putative target genes that were suspected to have multiple, substantially different hits were excluded. These included genes for which samples consistently recovered multiple hits and those hits contained regions that were difficult to align, as well as genes for which samples consistently recovered multiple hits, and the hits aligned without major gaps, but individual hits from one sample exhibited higher sequence similarity and identity to individual hits from other samples (often >80% AA sequence similarity and identity, measured in Sequence Manipulation Suite 3 [SMS3; Stothard, 2000]) than the other hits from that sample (often <60% AA sequence similarity and identity). These putative targets were considered to have a higher risk of targeting multiple distinct genes or copies that would produce additional challenges for assembly of recovered reads. In contrast, genes retained as targets contained one or two hits from samples, and those hits exhibited similar AA sequence similarity and identity (typically >90% within *Potamogeton* and >80% across Potamogetonaceae) to both hits from the same sample as well as hits from other samples. For these alignments, the multiple hits were considered to be more consistent with homeologs or allelic variation.

Consensus sequences were generated from adjusted alignments of target genes, and those consensus sequences were used for generation of a myBaits Custom Hybridization Capture Kit (Daicel Arbor Biosciences, Ann Arbor, Michigan, USA). At Daicel Arbor Biosciences, targets were softmasked for simple and low‐complexity repeats against the plant repeat database. As no Potamogetonaceae reference genome is publicly available, all bait candidates were BLASTed against genomes of *Phyllospadix torreyi* S. Watson, *Zostera marina*, *Posidonia oceanica* (L.) Delile, *Cymodocea nodosa* Asch., and *Thalassia testudinum* Banks & Sol. ex K.D. Koenig to identify those corresponding high‐copy genes or repetitive elements in the genomes. Bait candidates were also BLASTed against organellar genomes for those species as well as the available *Potamogeton crispus* chloroplast genome to remove any baits corresponding to organellar genes. In total, 19,696 baits were designed using 120‐nucleotide probes and approximately 3× tiling, resulting in a probe every ~35 nucleotides.

### Sample collection, library preparation, and target enrichment

Field samples were collected from populations of *Potamogeton* and *Stuckenia* across the eastern United States. Leaf tissues from each field sample were collected and dried in silica prior to DNA extraction. Tissue from each sample was homogenized using the FastPrep‐24 bead lysis system (MP Biomedicals, Irvine, California, USA). DNA was extracted from the dried field tissues following the protocol of Štorchová et al. (2000), modified for overnight incubation during cell lysis and DNA precipitation. Herbarium samples were collected from the Steere Herbarium of the New York Botanical Garden (Appendix S3). Qiagen DNeasy Plant Mini Kits (Qiagen, Hilden, Germany) were used to extract DNA from herbarium samples. Extractions followed the manufacturer's protocol, modified for overnight incubation during cell lysis. Following extraction, DNA fragment sizes were visualized on a 1% agarose gel, and DNA was quantified with Qubit dsDNA BR or HS Assay Kits using an Invitrogen Qubit 2.0 Fluorometer (Thermo Fisher Scientific, Waltham, Massachusetts, USA). The DNA was cleaned and concentrated prior to library prep using the Serapure bead solution of Faircloth and Glenn (2011).

Genomic libraries were prepared using NEBNext UltraExpress DNA Library Prep Kits according to the manufacturer's protocol (New England Biolabs, Ipswich, Massachusetts, USA). We used an increased volume of beads (1.2× reaction volume) during the final sample cleanup to preserve DNA fragments as small as 200 bp (verified by gel electrophoresis). Cleaned libraries were quantified again using the Qubit Fluorometer and combined into pools of 4–12 samples based on DNA concentration prior to target enrichment. Pools for samples of higher concentration were larger (10–12 samples), while samples of lower concentrations were combined into smaller pools (4–6 samples). Herbarium and silica‐dried tissues were pooled together according to concentration. The total DNA in a pool ranged from approximately 1500 ng to as low as 10 ng in pools of more heavily degraded samples. Samples belonging to *Stuckenia* and taxa outside of Potamogetonaceae were pooled separately from samples of *Potamogeton*.

For each pool, 7 µL was used as input for target enrichment reactions; the remaining volume of unenriched, pooled library was used to generate genome skim data for high‐copy regions like chloroplast and nuclear ribosomal sequences. Target enrichment was performed with the myBaits Custom DNA‐Seq kit from Daicel Arbor Biosciences using the standard protocol with a half volume of baits. Hybridization occurred for 36 h and enriched pools were amplified for 12 PCR cycles after hybridization. DNA concentration of the pools was quantified using the Qubit Fluorometer before and after target enrichment to ensure amplification of target sequences. Pooled libraries were sent to the University of Georgia Genomics and Bioinformatics Core (https://dna.uga.edu/) for quality control and sequencing in an Illumina NextSeq 2000 (Illumina, San Diego, California, USA). Unenriched and enriched libraries were sequenced in two separate runs, with the unenriched run on a P1 Flow Cell (300 cycles, 150‐bp paired‐end) and the enriched run on a P2 Flow Cell (300 cycles, 150‐bp paired‐end). Reads for the two sequencing runs were processed through separate pipelines.

### Genome skim data

To assemble chloroplast, ITS, and 5S‐NTS sequences from unenriched libraries, we used Captus v1.2.0 (Ortiz et al., 2026). Reads were cleaned using the default settings for quality trimming and specifying the Illumina adapter set. Cleaned reads were quality checked using Falco (de Sena Brandine and Smith, 2021). Contigs were assembled with default settings for minimum contig count and contig depth. Available chloroplast genomes for *Heteranthera dubia* (Jacq.) MacMill., *Najas flexilis* (Willd.) Rostk. & W.L.E. Schmidt, five species of *Potamogeton*, *Stuckenia pectinata*, and *Zannichellia palustris* were used to generate target files to extract assembled chloroplast data (Appendix S2). Chloroplast genes were extracted from available chloroplast genomes in Geneious by filtering for annotations of type “gene”; extraction used the default settings for minimum coverage and depth of extracted sequences. Extracted assemblies were aligned and trimmed using the default tools and settings in Captus (i.e., MAFFT *mafft_auto* [Katoh and Standley, 2013] and ClipKIT gappy [Steenwyk et al., 2020], respectively). Trimmed alignments corresponding to the matched sequence region that utilized naive filtering and included reference sequences were concatenated in Geneious; the concatenated alignment served as the input for gene tree inference (see below).

To extract assembled nuclear ribosomal regions, target sequences for the ITS region (spanning the 18S, 5.8S, and 28S ribosomal genes and both internal transcribed spacers), as well as sequences for the 5S‐NTS locus, were obtained from available data on GenBank (Appendix S4). For 5S‐NTS, trimmed, unfiltered alignments with references, including the matched sequences and the flanking regions, were used for gene tree inference. The initial extraction using the target ITS and 5S‐NTS sequences failed to recover multiple haplotypes for several known hybrid samples from which haplotypes had been recovered via cloning (Ramm and Frost, unpublished data). The *merge_level* threshold was increased to 0.99 to prevent highly similar haplotypes from being assembled into the same contig, but assembly still failed to recover both parental haplotypes for known hybrids. To attempt to recover all ITS haplotypes for comparison with previously generated data, the 5.8S gene and flanking ITS regions were extracted from the full‐length ITS target sequence for *Stuckenia pectinata*. Assemblies with the higher *merge_level* threshold were BLASTed against the ITS target. BLAST hits were extracted, combined with available data from GenBank (Appendix S4), and aligned with MAFFT using the “localpair” setting before gene tree estimation.

Gene trees were estimated for ITS, 5S‐NTS, and the concatenated chloroplast genes using IQ‐TREE 2 with ModelFinder to estimate nucleotide substitution models for each locus (Kalyaanamoorthy et al., 2017; Minh et al., 2020). Branch support values were estimated using 5000 ultrafast bootstrap replicates with nearest neighbor interchange (Hoang et al., 2018), as well as 5000 Shimodaira–Hasegawa‐like approximate likelihood ratio test (SH‐aLRT) replicates (Guindon et al., 2010). The inferred relationships of recovered haplotypes were used to evaluate the performance of these loci that have typically been used in previous studies to that of our probe set for hybrid detection.

### Target‐enriched data

Sequence reads from target‐enriched libraries were trimmed using Trimmomatic v0.39 (Bolger et al., 2014) on default settings using the TruSeq3‐PE‐2.fa adapter file. We used default settings for quality trimming, a minimum read length of 24 bp, and the keepBothReads option set to true. Trimmed reads were assembled into supercontigs using HybPiper2 v2.3.2 (Johnson et al., 2016). To improve assembly for outgroup samples, we used a custom target file composed of consensus sequences for each locus for various species throughout Alismatales. Default settings for read depth and coverage were used. Twelve additional accessions of *Stuckenia*, *Zannichellia*, *Zostera*, and *Phyllospadix* Hook. (Zosteraceae) obtained from GenBank were included for assembly.

The HybPhaser workflow v2.1 (Nauheimer et al., 2021) allows for the detection of putative hybrids and subsequent phasing of reads into multiple accessions using a collection of Bash and R scripts (https://github.com/LarsNauheimer/HybPhaser). First, consensus sequences were generated by mapping each sample's sequence reads to the assembled contigs of each locus. Ambiguity codes were assigned at sites containing single nucleotide polymorphisms (SNPs) with a minimum read depth of 10 and an alternate nucleotide appearing in at least 15% of reads. Following the recommendations of Nauheimer et al. (2021), genes that had sequences recovered for less than 20% of samples or an average less than 25% of the target sequence length recovered were removed from the dataset. In addition, all samples with less than 20% of genes recovered or less than 45% of the target sequence length recovered were removed. Finally, genes with outlier proportions of SNPs were flagged as potential paralogs and removed as well. Note that, for simplicity and consistency, this and future use of “paralogs” follows the operational use of the word by assembly pipelines. HybPhaser summary statistics for each sample were collated into a summary table (Appendix S5).

Multiple sequence alignments for each gene were generated from the ambiguity‐coded consensus sequences for each sample and aligned using MAFFT v7.525 (Katoh and Standley, 2013). Maximum‐likelihood gene trees were estimated for each locus using IQ‐TREE v2.3.2 with ModelFinder (Kalyaanamoorthy et al., 2017; Minh et al., 2020). Branch supports for preliminary trees were estimated using 1000 ultrafast bootstrap replicates with nearest neighbor interchange search (Hoang et al., 2018). Gene trees were summarized into a coalescent species tree using wASTRAL v1.22.3.7 (Zhang and Mirarab, 2022) with default settings. This species tree and the preliminary gene trees were used to determine suitable reference samples for clade association. Clade references were selected as samples with high data recovery (Appendix S6), low measures of locus heterozygosity and allele divergence (Appendix S5), and consistent placement in preliminary trees.

In total, 36 samples were selected as references for clade association, representing clades for 31 species of *Potamogeton*, four species of *Stuckenia*, and a single species of *Zannichellia*. On‐target reads were mapped simultaneously to each reference using BBSplit (https://sourceforge.net/projects/bbmap), and the percentage of reads mapping unambiguously to each was recorded in a summary table. Accessions of three confirmed hybrids (*P*. ×*absconditus* Z. Kaplan, J. Fehrer, and C.B. Hellquist, *P*. ×*floridanus* Small, and *P*. ×*mysticus* Morong) were used to compare various putative hybrid scenarios during clade association. These three hybrids each have parental combinations of different phylogenetic distances and helped to clarify the expected associations of hybrids with both closely related and distantly related parent species. Samples were phased if they had significant proportions of reads associated with multiple clade references (Appendix S7). Phased accessions were assembled in HybPiper and processed in the first part of HybPhaser. To avoid discarding only one accession of a phased pair, phased accessions were not filtered based on locus recovery. The same genes removed in the first analysis were also removed for all phased accessions. Consensus sequences were generated for phased accessions and combined with those of non‐phased samples for phylogenetic analysis.

Consensus sequences for the combined dataset were aligned using MAFFT (Katoh and Standley, 2013) and cleaned with ClipKIT v2.4.1 (Steenwyk et al., 2020) using the default smart‐gap trimming mode. Summary statistics for each locus were calculated with AMAS (Borowiec, 2016). Maximum‐likelihood gene trees were estimated for each locus again using IQ‐TREE 2 with ModelFinder (Kalyaanamoorthy et al., 2017; Minh et al., 2020). Branch supports were estimated using 5000 ultrafast bootstrap replicates with nearest neighbor interchange (Hoang et al., 2018). Outlier long branches were removed from gene trees using TreeShrink with default settings (Mai and Mirarab, 2018). Gene trees were summarized into a coalescent species tree using wASTRAL (Zhang and Mirarab, 2022) with the hybrid weighting scheme (i.e., quartet scores are optimized by weighting branch length and statistical support of a branch in input gene trees). Tree search was performed with 16 rounds of tree search and subsampling and branch supports estimated by local posterior probabilities (LPP).

## RESULTS

### Sequencing and assembly

De novo assembly of off‐target data with Captus resulted in contigs for 285 of 288 samples; assembly statistics are provided in Appendix S8. After filtering, an average of 5216 contigs remained per sample (6–49,025 contigs). Most assembled contigs were short: the average length of contigs across samples was 370 bp (143–639 bp average length), and the percentage of contigs greater than 1 kbp was low, with an average of 1.7% and a maximum of 12.6%. Average read depth for contigs was 12.3, ranging from 3.0× to 74.9× coverage. After recognizing the need for short target sequences to most effectively extract sequences, we extracted contigs for all chloroplast genes with an average of 205 (128–246) samples represented in each gene. The initial concatenated alignment included 276 of the 285 samples that had assembled contigs. After removing samples with poor data recovery, the final concatenated cpDNA alignment comprised 233 sequences, 224 generated in this study and nine reference sequences.

### Performance of the Pondweeds350 probe set for target enrichment

Our novel probe set, Pondweeds350, targets 350 loci in total, combining 132 custom Potamogetonaceae targets, 205 Angiosperms353 loci, and 13 functional genes for aquatic adaptation. The probes comprise 737,266 bp across the 350 genes, averaging 2466 bp (414–14,259 bp) per gene. Excluding the five samples that failed completely, the remaining samples had an average of 315 (range: 1–347) genes recovered at 50% target length and an average of 296 (range: 0–339) genes recovered at 75% target length (Appendix S6). Herbarium specimens performed slightly better on average than silica‐dried collections, with an average of 301 genes recovered at 75% compared to 287 for the field collections. Across all samples, there was an average of 349 (range: 323–350) genes with reads mapped, an average of 336 (range: 12–350) genes producing contigs, and an average of 327 (range: 6–350) genes producing sequences (Appendix S6). With 737,266 targeted base pairs, we recovered an average of 635,500 bp per sample, or 86.2% of the targeted length. The initial HybPiper assembly issued long paralog warnings for 233 and paralog warnings by depth for 248 of the 283 samples. The number of long paralog warnings ranged from 1 to 69, with an average of 12 long paralogs for samples that received warnings. Paralog‐by‐depth warnings ranged from 1 to 321, with an average of 82 paralogs for samples that received warnings. Samples identified as hybrids generally did not receive more warnings than samples of discrete species.

After filtering based on target sequence length and number of samples, 323 loci and 269 samples remained. After phasing 36 samples and combining with the 233 unphased samples, the number of assemblies totaled 305. The ClipKit‐trimmed loci contained an average of 295 sequences (100–314 sequences) and were, on average, 8759 bp (2113–36,180 bp; Appendix S9). Alignments contained an average of 46% variable sites and 26% parsimony informative sites, with an average of 58.0% missing data (28.0–80.0%). Comparing custom targeted genes for Potamogetonaceae to genes that are also included in the Angiosperms353 probe set, the two sets of loci performed similarly in terms of the average number of samples per alignment (296 and 294, respectively). A higher proportion of Angiosperms353 loci passed the filtering criteria (96.6%) than did the custom Potamogetonaceae loci (88.6%). In addition, the Angiosperms353 loci had slightly higher proportions of variable and parsimony informative sites (0.48 and 0.28, respectively) compared to the custom loci (0.44 and 0.22). However, the custom loci were also on average significantly longer (average: 12,099 bp) than the Angiosperms353 loci (average: 6802 bp).

### Hybrid detection

#### HybPhaser

Prior to phasing, HybPhaser filters out samples and loci with poor data recovery. Twelve samples fell below the 20% threshold for recovered loci; these 12 and an additional 19 samples also fell below the 45% threshold for target sequence length recovered. All 31 of these samples were excluded from further analysis. In addition, six loci were removed due to being recovered in less than 20% of samples. Finally, 20 loci were flagged by HybPhaser as containing outlier proportions of SNPs across all samples, which HybPhaser uses to indicate potential paralogs. One additional gene that was not filtered out was later excluded from the phylogenetic analysis because it contained less than 25% of all samples. The resulting dataset comprised 323 loci across 269 samples. Excluding the outgroup taxa, an average of 317 loci and a total sequence length of 1.23 million base pairs were recovered per sample. The average locus heterozygosity and allele divergence across all samples were 88.8% and 2.58%, respectively (Appendix S5). Significantly higher values were observed for polyploid taxa as well as some known and putative hybrids; these were typically crosses that involved polyploid species (Figure 2, Appendix S5). Additionally, the highest measures of allele divergence were seen in samples of *Stuckenia*, which are all hexaploid (Kaplan et al., 2013; Fehrer et al., 2022b).

**Figure 2:**
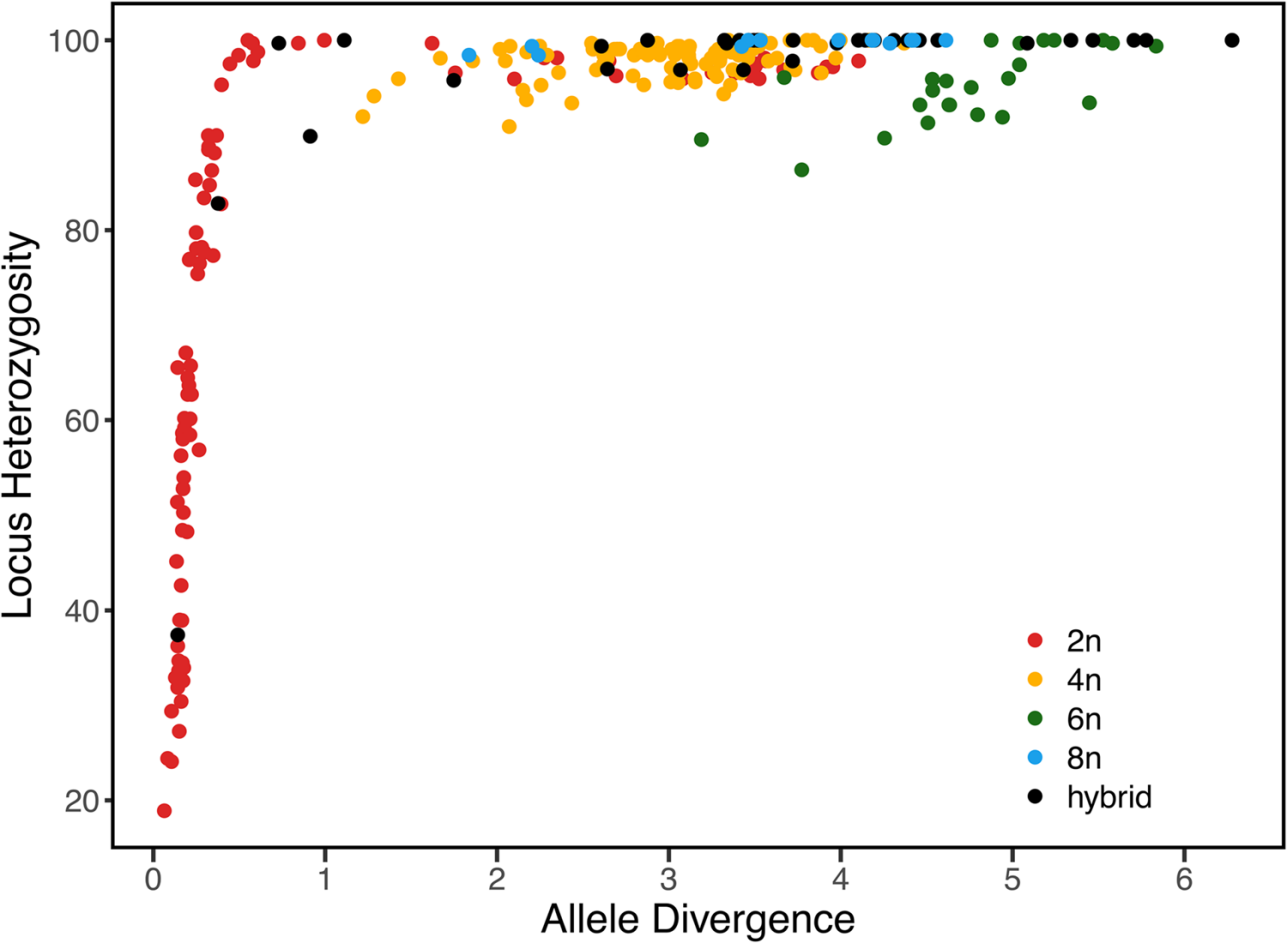
Allele divergence (%) versus locus heterozygosity (%) for accessions analyzed in this study. The samples are plotted by known/expected ploidy to show that these values generally increase with ploidy.

One clade reference was selected per species, with the exception of the *Potamogeton spirillus* group. This group currently comprises three accepted species (*P. spirillus* Tuck., *P. diversifolius* Raf., and *P. bicupulatus* Fernald) but contained four well‐supported subclades in the preliminary phylogeny; one reference was selected per clade. Hybrid samples are expected to have high proportions of reads mapping unambiguously to multiple clade references, reflecting the inheritance of alleles from different species. However, the percentage of reads that map unambiguously is a function of how closely related references are to each other as well as the evolutionary history of the group (Nauheimer et al., 2021; Bloesch et al., 2022). The percentages of unambiguously mapping reads were generally low, even when comparing a reference to itself. Most samples had approximately 5–10% of their reads mapped to the reference for their species but less than 2% mapping to any other species.

Thirty‐six samples were considered to have significant proportions of reads associated with multiple references and were phased as putative hybrids (Appendices S5, S10). A total of 26 unique hybrid combinations were detected, 16 of which represent undescribed hybrids. Of the 36 samples phased, 30 had greater than 2.5% allele divergence and 95% locus heterozygosity (Figure 2, Appendix S5). For the remaining six phased samples, they either had significantly less data recovered than their counterparts or are determined to be hybrids between closely related diploid species. Generally, phased accessions had similar proportions of reads that were unambiguous to each reference after phasing; however, in hybrid crosses of species with different ploidy levels, we observed ratios that were inversely proportional to the ploidy ratio (i.e., roughly double the reads matching a diploid parent compared to a tetraploid parent; Appendix S11). This is most likely attributable to polyploid species having greater proportions of SNPs overall (Appendix S5), and thus fewer reads map unambiguously.

#### Genome skim data

To explore the utility of the off‐target loci for hybrid detection, the number of haplotypes recovered and relationships of the haplotypes were examined for the 36 phased samples. All 36 samples had data for 5S‐NTS, 32 had data for ITS, and 31 had data for cpDNA. For ITS, multiple haplotypes were recovered for 17 of the 32 samples with data, and the maximum number of haplotypes recovered for a sample was five (average: ~2 haplotypes). For 5S‐NTS, 31 of the phased samples recovered multiple haplotypes, and the maximum number of haplotypes for a sample was 47 (average: ~7 haplotypes). Acknowledging the limitations in our assembly of these loci from genome skim data, one novel hybrid combination remained undetectable across all three loci: *P. epihydrus* Raf. × *P. tennesseensis* Fernald.

### Phylogenetic analysis

Our tree inferred from concatenated cpDNA resolved both *Potamogeton* and *Stuckenia* as monophyletic (Figure 3, Appendix S12). Support along the backbone, for major subclades, and for species‐level clades was generally high (>95/>70 SH‐aLRT/ultrafast bootstrap support). Phylogenetic results for the ITS region were generally poorly supported along the backbone but with high support for some subclades and species‐level clades. The ITS topology (Appendix S13) recovered a monophyletic Potamogetonaceae with high support (97.9/99) and monophyletic genera for *Stuckenia*, *Groenlandia*, and *Zannichellia*. However, subclades in *Potamogeton* and relationships among them were poorly supported in the ITS tree. For the 5S‐NTS tree (Figure 4, Appendix S14), monophyletic genera were recovered for *Potamogeton* and *Stuckenia* with high support (100/100), and species within subclades were mostly consistent with other analyses. The four subclades of the broad‐leaved clade were resolved as monophyletic with low support (89.5/92), with *P. robbinsii* Oakes and *P. crispus* (B1) as sister to the rest of the broad‐leaved clade. The linear‐leaved clade was also resolved as monophyletic, albeit with weak support (9.88/88).

**Figure 3:**
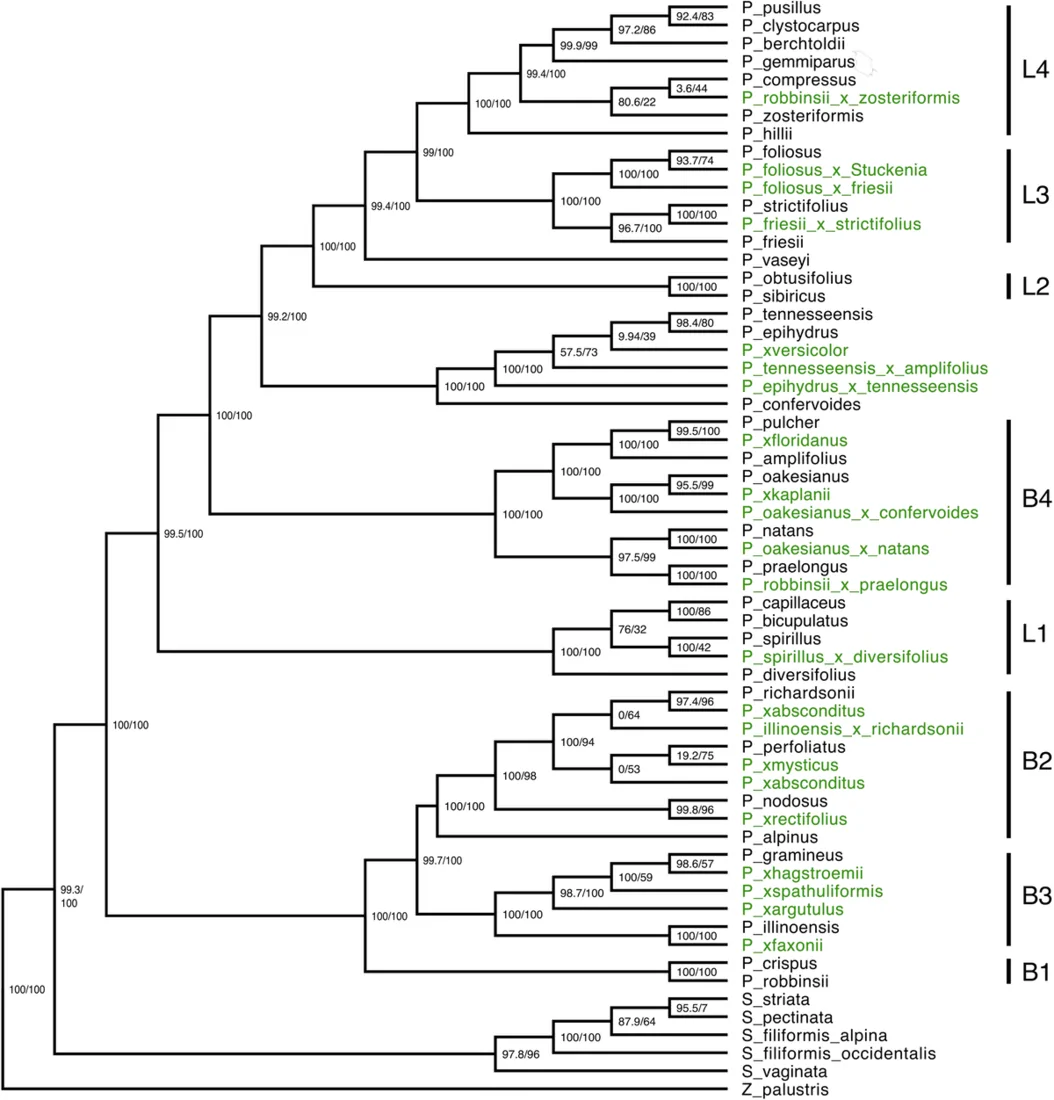
Simplified topology for cpDNA tree for North American pondweeds. Hybrid samples are colored green. Support values at nodes reflect Shimodaira–Hasegawa‐like approximate likelihood ratio test (SH‐aLRT)/ultrafast bootstrap support. Clade labels correspond to subclades defined by Ito et al. (2016); clades labeled with an “L” denote linear‐leaved subclades and clades labeled with a “B” denote broad‐leaved subclades.

**Figure 4:**
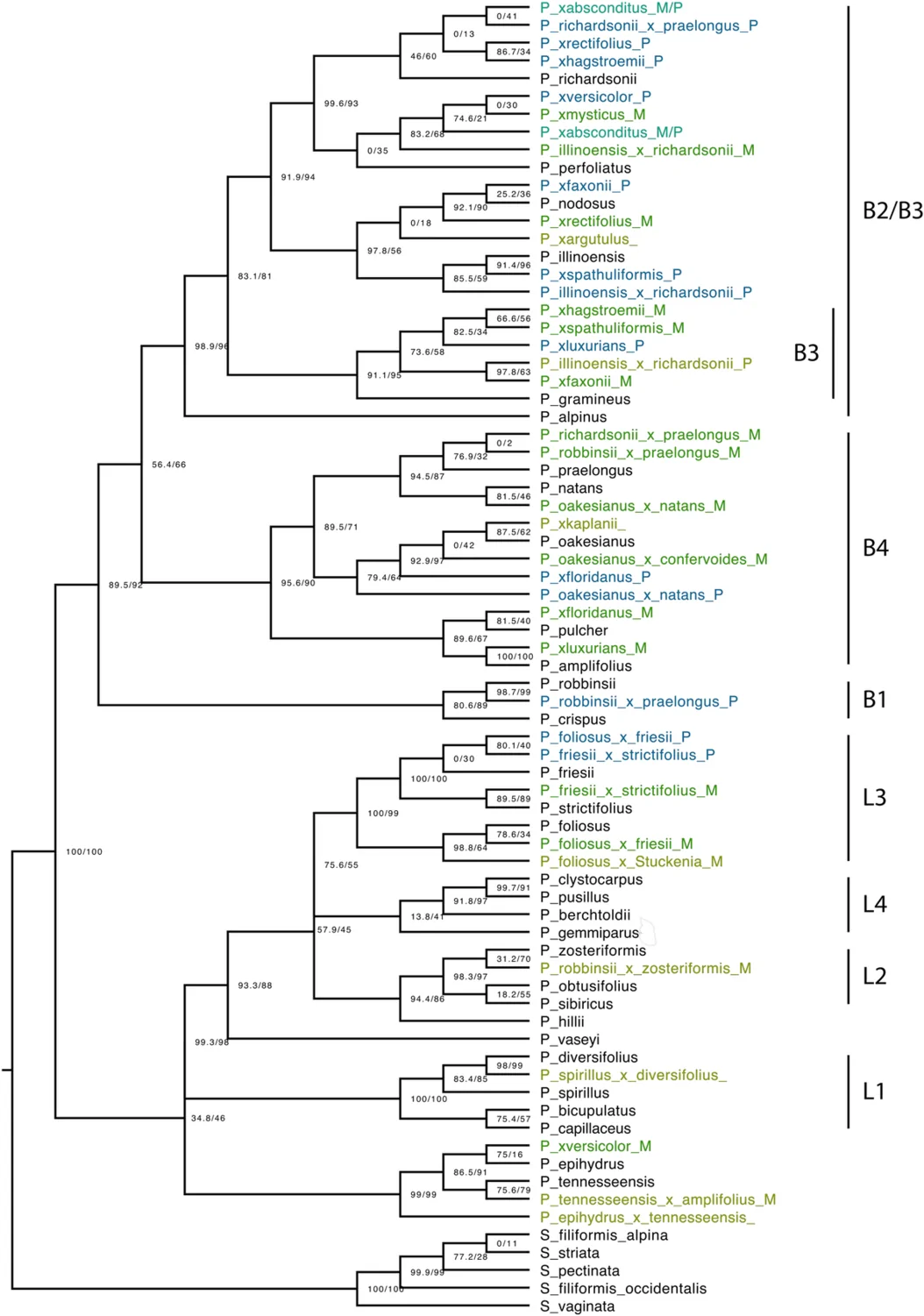
Simplified topology for 5S non‐transcribed spacer (5S‐NTS) tree of North American pondweeds. Phased hybrid samples are labeled and colored for maternal (M; green) and paternal (P; blue) haplotypes. Hybrids for which only one haplotype was recovered are colored yellow, and those for which the cross is inferred to have occurred in both directions are colored cyan. Support values at nodes reflect SH‐aLRT/ultrafast bootstrap support. Clade labels correspond to subclades defined by Ito et al. (2016); clades labeled with an “L” denote linear‐leaved subclades and clades labeled with a “B” denote broad‐leaved subclades.

The overall topology of our coalescent species tree (Figure 5) was similar to the 5S‐NTS tree. *Stuckenia* was again resolved as a monophyletic group distinct from *Potamogeton*. Most discrete species of *Potamogeton* form fully supported clades. We found full support for the division between the broad‐leaved and linear‐leaved clades, with *P. crispus* and *P. robbinsii* (B1) weakly supported as sister to both clades (0.70 LPP). Within the overall broad‐leaved clade, the B4 subclade was fully supported as sister to the other broad‐leaved species. *Potamogeton nodosus* Poir., *P. perfoliatus*, *P. richardsonii* (A. Benn.) Rydb., and *P. alpinus* formed a moderately supported subclade, with *P. alpinus* placed sister to the rest of the subclade (B2, 0.98 LPP). *Potamogeton gramineus* and *P. illinoensis* Morong (B3) together form a grade to B2. Although the combined B2/B3 subclade lacks support (0.5), the broad‐leaved clade including B2/B3 and B4 is fully supported. We additionally found high support (0.99–1 LPP) for each of the described linear‐leaved subclades. *Potamogeton epihydru*s, *P. tennesseensis*, and *P. confervoides* Rchb., which were unplaced in Ito et al. (2016), were included in the greater linear‐leaved clade but resolved as separate from any of the defined subclades.

**Figure 5:**
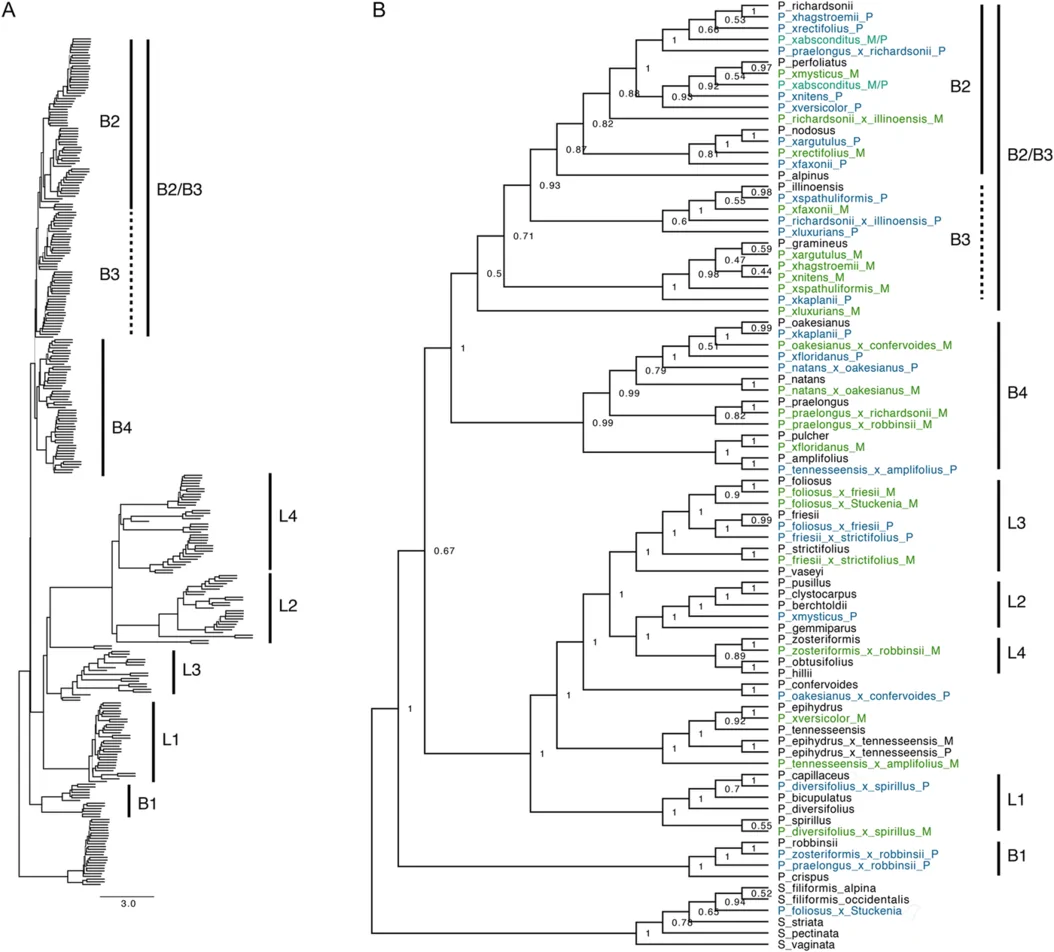
wASTRAL results for samples of Potamogetonaceae. (A) Full wASTRAL topology with branches weighted by support values (local posterior probability [LPP]). (B) Simplified wASTRAL cladogram with one representative sample per species. Support values at nodes reflect branch support in wASTRAL (LPP). Phased hybrid samples are labeled and colored for maternal (M; green) and paternal (P; blue) haplotypes. Clade labels correspond to subclades defined by Ito et al. (2016); clades labeled with an “L” denote linear‐leaved subclades and clades labeled with a “B” denote broad‐leaved subclades.

## DISCUSSION

### Performance of the Pondweeds350 probe set

Robust phylogenetic inference for Potamogetonaceae, particularly *Potamogeton*, has historically been challenging (Lindqvist et al., 2006; Ito et al., 2016). With Sanger sequencing, researchers struggled to identify loci with sufficient variation to resolve relationships (Figure 1). Hyb‐Seq with high‐throughput sequencing methods offers efficient capture of hundreds to thousands of nuclear loci and the opportunity to improve resolution for difficult clades. Universal probe sets like Angiosperms353 allow the collection of these data without the development and expense of custom probes; however, success varies across lineages (Johnson et al., 2019). For example, the representative sample for Alismatales—*Butomus umbellatus* (Butomaceae)—exhibited low enrichment efficiency (18.2% of reads mapping to a target sequence) and recovered relatively few (95) genes at 50% of the target length in the initial efficacy tests of Angiosperms353 (Johnson et al., 2019, but note that assembly was improved with a custom target file [McLay et al., 2021]). Combining custom targeted genes with clade‐specific targets for genes from universal probe sets offers the advantages of efficiency and efficacy for the group of interest, as well as the ability to incorporate data generated with universal probe sets. With the understanding that sequence variability was low in Potamogetonaceae among loci targeted by universal primers and Sanger sequencing in the past and the need for effective enrichment in order to phase parental copies from hybrid accessions, we elected to develop a custom probe set with Potamogetonaceae‐specific target sequences for 205 Angiosperms353 loci as well as 132 custom loci and 13 functional genes for aquatic lifestyles. Our Pondweeds350 probe set marks an advancement for both phylogenetic inference and hybrid detection in Potamogetonaceae.

We demonstrate the success of the probe set, with high capture efficiency and strong recovery across samples and loci (Appendix S8). Using primarily herbarium samples—179 of the 288 samples, some dating as far back as 1864—only five failed to recover sequence data, and 24 were eliminated for insufficient data. Additionally, an average of 296 genes were recovered at 75% target length in successful samples (Appendix S8). Because our dataset did include a high proportion of herbarium samples, many samples in our dataset had low starting concentrations of DNA. For example, 72 enriched libraries had concentrations <5 ng/µL, with total DNA <140 ng prior to pooling. Among the lowest of these (five samples with <1 ng/µL and <20 ng total DNA), four of the samples, including a known hybrid sample, successfully recovered data for at least 341 total genes and 206 genes at least 50% of the target length. This success with degraded DNA at low concentrations highlights not only the efficacy of the Pondweeds350 probe set but also the utility of herbarium specimens for these analyses.

### Systematics of North American pondweeds

We provide the most densely sampled and well‐resolved phylogeny of North American Potamogetonaceae to date. We show strong support for the monophyly of the broad‐leaved and linear‐leaved clades, with mixed results regarding the subclades defined by Ito et al. (2016) (Figures 1 and 5). Moreover, our combined analysis strongly supports the monophyly of most described species of *Potamogeton* in North America. However, the status of some species remains unresolved even with the addition of more data.

The L1 subclade (subsection *Hybridi* in Fernald, 1932) contains three currently accepted species: *P. spirillus*, *P. diversifolius*, and *P. bicupulatus*. These three species are nearly morphologically identical when not in flower and have historically been distinguished primarily by geography. However, our results support an additional taxon within this clade resolved as sister to *P. bicupulatus* (Figure 5). The fruit morphology of specimens belonging to this taxon is distinct from the other three taxa and closely resembles the species *P. capillaceus* Poir. based on Fernald's account of the species (Fernald, 1932). Its position in our phylogeny also matches its taxonomic position in Fernald's key as the sister taxon to *P. bicupulatus*. This taxon is no longer accepted after Reznicek and Bobbette (1976) found that the morphology of the type specimen did not match Fernald's description. Here, we have reported this taxon as *P. capillaceus*; however, a more thorough analysis of this clade is necessary to determine its specific status.

Similarly, the L4 subclade has a complicated history based on limited morphological differences between taxa. Here, we recognize four phylogenetically distinct taxa within the clade: *P. pusillus*, *P. berchtoldii*, *P. gemmiparus* J.W. Robbins ex Morong, and *P. clystocarpus* Fernald (Figure 5). Les et al. (2009) determined *P. gemmiparus* and *P. clystocarpus* to be regional subspecies of *P. berchtoldii* based on the very limited ranges of these taxa. However, in our analysis these three are not monophyletic with respect to *P. pusillus*. Greater sampling of this clade is likely needed to support or reject the species status of *P. berchtoldii*, *P. gemmiparus*, and *P. clystocarpus*.

Additionally, while we were able to clearly detect 16 novel hybrids, there appear to be several taxa with more complicated histories that are currently unclear. One of the novel hybrids (voucher MHN59347, NYBG:2431048) was phased against three references as a triple hybrid following relationships indicated by the off‐target data (Appendices S12–14). Although there is evidence for at least one triple hybrid in *Potamogeton* (Kaplan and Fehrer, 2007), they are assumed to be very rare, as hybrid pondweeds are broadly observed to be sterile (Kaplan et al., 2009; Kaplan and Fehrer, 2006, 2013). This triple hybrid presumably would result from an initial hybridization between *P. richardsonii* and *P. perfoliatus* (which produce the fertile hybrid *P*. ×*absconditus*), followed by a second hybridization with *P. illinoensis*.

Lastly, more work is needed to clarify the status of *P. illinoensis* and the genus sister to *Potamogeton*, *Stuckenia*. *Potamogeton illinoensis* is morphologically and genetically similar to both *P. nodosus* and *P. gramineus* and, based on phylogenetic discordance in our preliminary trees, is potentially of hybrid origin (Appendices S12–14). During clade association, accessions of *P. illinoensis* had higher associations with the references for both of those species than with the *P. illinoensis* reference (Appendix S10); however, phasing did not improve phylogenetic resolution, and these samples are not counted among our 36 putative hybrids. Similarly, *Stuckenia* seems to have undergone extensive hybridization and likely introgression as well. No samples of *Stuckenia* were phased as the clade association indicated gene flow between most species of *Stuckenia* sampled and clear hybrid taxa could not be determined (Appendix S10). Despite this, there was evidence of intergeneric gene flow between one sample of *Potamogeton foliosus* Raf. and an undetermined species of *Stuckenia*.

Challenges still remain for phylogenetic inference of Potamogetonaceae given the complexity in the dataset arising from polyploidy and frequent hybridization. At the time of probe design and manufacturing, there were no assembled genomes available. There have since been reference genomes for nine species of *Potamogeton* and one for *Stuckenia pectinata* published by the Wellcome Sanger Institute (Hinxton, United Kingdom) added to NCBI's Genome resources (https://www.ncbi.nlm.nih.gov/datasets/genome/?taxon=16362). While our approach of BLASTing Sondovač targets back to transcriptomes in order to target full‐length genes provided efficient assembly of separate targets corresponding to the same gene—accommodating assumptions of phylogenetic analyses—it also introduced the potential for assembly of chimeric sequences (i.e., one supercontig composed of contigs from separate copies). Using the consensus sequences output by HybPhaser for discrete species and phased reads from hybrids reduced the impact of chimeric sequence assembly, because there was not an attempt to assemble individual paralogs. However, conflicting phylogenetic signals and the presence of copies inherited through duplication rather than speciation remained. The hypothesis for North American Potamogetonaceae presented here marks an advancement in the understanding of phylogeny and taxonomy of pondweeds. Future work aims to utilize the now‐available reference genomes to improve reference sequences for assembly as well as to identify and filter paralogs.

### Hybrid identification in Potamogetonaceae

The results of HybPhaser's clade association step reflected the patterns observed in the ITS, 5S‐NTS, and chloroplast trees (Appendices [Link], [Link]–S14). Furthermore, the results of known hybrid accessions during clade association and phasing were consistent with our expectations based on known parental relationships and show that the HybPhaser workflow is suitable for detecting and phasing hybrids in Potamogetonaceae. Despite similar effectiveness in hybrid detection, the use of target sequence capture and the HybPhaser workflow has several advantages over prior studies. One of the advantages is the elimination of the time‐consuming process of cloning unique haplotypes to determine parentage. Moreover, the workflow does not require an existing hypothesis of the parentage to dictate sampling. Predicting parentage based on morphology is not straightforward in Potamogetonaceae, as some hybrids are so morphologically similar to one parent that it can be unclear whether a population is of hybrid origin or simply an unusual phenotype. For taxa that have highly similar morphology, a hybrid with an intermediate phenotype can easily be misidentified as one of the two parent species, as is the case for our putative hybrids between *P. natans* and *P. oakesianus* J.W. Robbins. Finally, for hybrid populations that do not co‐occur with their parent species, the correct parentage may be difficult to determine based on geographic ranges, such as in the case of *P. ×floridanus* (Kaplan et al., 2018).

Our Potamogetonaceae‐specific probe set is a novel tool that provides an effective way to generate target capture data for many pondweed samples with limited wet‐lab time required per sample. The use of target sequence capture and high‐throughput sequencing of numerous loci allows data generation for hundreds of loci and for hundreds of samples, enabling us to focus on broad geographic and taxonomic sampling. Due to our sampling pattern, we were able to infer a more comprehensive species‐level phylogeny than previous studies, while also easily detecting misidentified collections. Ultimately, our strategy enables the detection of hybrids that may otherwise remain unidentified, allowing hybrid detection in Potamogetonaceae at a scale not previously possible. While a deeper phylogenetic analysis of Potamogetonaceae is required to better understand the complex evolutionary history of the group, it is clear that hybridization is even more frequent in pondweeds than previously described. While the extent of hybridization in pondweeds remains unknown, our findings highlight both the utility and the need for specialized molecular tools to explore these scenarios. Future studies of Potamogetonaceae should focus on exploring methods to integrate hybrids into phylogenetic analyses, testing for scenarios of ancient hybridization, and exploring the conditions that lead to such extreme hybridization in North American pondweeds.

## AUTHOR CONTRIBUTIONS

L.A.F. conceptualized the study, supervised the research, and designed the probe set. Z.T.R. performed the molecular work and the hybrid identification. Both authors contributed to the phylogenetic analysis. Both authors wrote the manuscript and approved the final version.

## Supporting information


**Appendix S1**: Transcriptomes and genome skims for Alismatales obtained from the NCBI Sequence Read Archive (SRA) for target capture probe design.
**Appendix S2**: Organellar genomic references used for target capture probe design and/or assembly of recovered cpDNA sequences.
**Appendix S3**: Voucher information for herbarium specimens used for target enrichment sampled from the Steere Herbarium of the New York Botanic Garden.
**Appendix S4**: GenBank accessions used to determine target sequences for the ITS and 5S‐NTS nuclear ribosomal loci.
**Appendix S5**: HybPhaser dataset optimization summary table.
**Appendix S6**: HybPiper assembly statistics.
**Appendix S7**: List of samples phased as putative hybrids.
**Appendix S8**: Captus assembly statistics.
**Appendix S9**: AMAS locus summary statistics.
**Appendix S10**: HybPhaser clade association results.
**Appendix S11**: HybPhaser phasing statistics.

**Appendix S12**: Full topology of concatenated cpDNA genes. Support values at nodes reflect SH‐aLRT/ultrafast bootstrap support.

**Appendix S13**: Full topology for the ITS nuclear ribosomal locus. Support values are omitted for readability.

**Appendix S14**: Full topology for the 5S‐NTS nuclear ribosomal locus. Support values are omitted for readability.

**Appendix S15**: Sequence Read Archive accession numbers for samples used in this study.

## Data Availability

Sequence reads are submitted to the Sequence Read Archive (SRA) under project PRJNA1428498 and project PRJNA1474426. SRA accession numbers are provided in Appendix S15. Alignment files, phylogenetic trees, and assembly statistics are available on Dryad (https://doi.org/10.5061/dryad.qfttdz0xj; Ramm and Frost, 2026).

## References

[aps370091-bib-0001] Álvarez, I. , and J. F. Wendel . 2003. Ribosomal ITS sequences and plant phylogenetic inference. Molecular Phylogenetics and Evolution 29: 417–434. 10.1016/S1055-7903(03)00208-2 14615184

[aps370091-bib-0002] Aykurt, C. , J. Fehrer , D. Sarı Yol , Z. Kaplan , V. Bambasová , İ. G. Deniz , E. Aydemir , et al. 2020. Taxonomic treatment and phylogenetic analysis of the family Potamogetonaceae in Turkey. TAXON 69: 1172–1190. 10.1002/tax.12364

[aps370091-bib-0003] Ball, L. D. , A. M. Bedoya , C. M. Taylor , and L. P. Lagomarsino . 2023. A target enrichment probe set for resolving phylogenetic relationships in the coffee family, Rubiaceae. Applications in Plant Sciences 11: e11554. 10.1002/aps3.11554 PMC10719880 38106541

[aps370091-bib-0004] Bloesch, Z. , L. Nauheimer , T. Elias Almeida , D. Crayn , and A. R. Field . 2022. HybPhaser identifies hybrid evolution in Australian Thelypteridaceae. Molecular Phylogenetics and Evolution 173: e107526. 10.1016/j.ympev.2022.107526 35577295

[aps370091-bib-0005] Bolger, A. M. , M. Lohse , and B. Usadel . 2014. Trimmomatic: A flexible trimmer for Illumina sequence data. Bioinformatics 30: 2114–2120. 10.1093/bioinformatics/btu170 PMC4103590 24695404

[aps370091-bib-0006] Borowiec, M. L. 2016. AMAS: A fast tool for alignment manipulation and computing of summary statistics. PeerJ 4: e1660. 10.7717/peerj.1660 PMC4734057 26835189

[aps370091-bib-0007] Camacho, C. , G. Coulouris , V. Avagyan , N. Ma , J. Papadopoulos , K. Bealer , and T. L. Madden . 2009. BLAST+: Architecture and applications. BMC Bioinformatics 10: e421. 10.1186/1471-2105-10-421 PMC2803857 20003500

[aps370091-bib-0008] Carpenter, S. R. , and D. M. Lodge . 1986. Effects of submersed macrophytes on ecosystem processes. Aquatic Botany 26: 341–370. 10.1016/0304-3770(86)90031-8

[aps370091-bib-0009] Chen, L.‐Y. , B. Lu , D. F. Morales‐Briones , M. L. Moody , F. Liu , G.‐W. Hu , C.‐H. Huang , et al. 2022. Phylogenomic analyses of Alismatales shed light into adaptations to aquatic environments. Molecular Biology and Evolution 39: msac079. 10.1093/molbev/msac079 PMC9070837 35438770

[aps370091-bib-0010] Christenhusz, M. J. M. , and J. W. Byng . 2016. The number of known plants species in the world and its annual increase. Phytotaxa 261: 201–217. 10.11646/phytotaxa.261.3.1

[aps370091-bib-0011] de Sena Brandine, G. , and A. D. Smith . 2021. Falco: High‐speed FastQC emulation for quality control of sequencing data. F1000Research 8: e1874. 10.12688/f1000research.21142.2 PMC7845152 33552473

[aps370091-bib-0012] Faircloth, B. C. , and T. C. Glenn . 2011. Faircloth Lab Serapure Bead Protocol. Website https://ethanomics.wordpress.com/wp-content/uploads/2012/08/serapure_v2-2.pdf [accessed 14 October 2026].

[aps370091-bib-0013] Fehrer, J. , S. Iida , and Z. Kaplan . 2022a. Cryptic species of pondweeds (Potamogetonaceae) at an intercontinental scale revealed by molecular phylogenetic analyses. TAXON 71: 531–551. 10.1002/tax.12686

[aps370091-bib-0014] Fehrer, J. , M. Nagy Nejedlá , C. B. Hellquist , A. A. Bobrov , and Z. Kaplan . 2022b. Evolutionary history and patterns of geographical variation, fertility, and hybridization in *Stuckenia* (Potamogetonaceae). Frontiers in Plant Science 13: e1042517. 10.3389/fpls.2022.1042517 PMC9670304 36407593

[aps370091-bib-0015] Fernald, M. L. 1932. The linear‐leaved North American species of *Potamogeton*, Section Axillares. Memoirs of the American Academy of Arts and Sciences 17: 1–183. 10.2307/25058195

[aps370091-bib-0016] Fu, L. , B. Niu , Z. Zhu , S. Wu , and W. Li . 2012. CD‐HIT: Accelerated for clustering the next‐generation sequencing data. Bioinformatics 28: 3150–3152. 10.1093/bioinformatics/bts565 PMC3516142 23060610

[aps370091-bib-0017] Guindon, S. , J.‐F. Dufayard , V. Lefort , M. Anisimova , W. Hordijk , and O. Gascuel . 2010. New algorithms and methods to estimate maximum‐likelihood phylogenies: Assessing the performance of PhyML 3.0. Systematic Biology 59: 307–321. 10.1093/sysbio/syq010 20525638

[aps370091-bib-0018] Han, X. , X. Chang , Z. Zhang , H. Chen , H. He , B. Zhong , and X. W. Deng . 2019. Origin and evolution of core components responsible for monitoring light environment changes during plant terrestrialization. Molecular Plant 12: 847–862. 10.1016/j.molp.2019.04.006 31009752

[aps370091-bib-0019] Haynes, R. R. , D. H. Les , and L. B. Holm‐Nielsen . 1998. Potamogetonaceae. In K. Kubitzki [ed.], Flowering plants. Monocotyledons: Alismatanae and Commelinanae (except Gramineae), 408–415. Springer, Berlin, Germany.

[aps370091-bib-0020] Hoang, D. T. , O. Chernomor , A. Von Haeseler , B. Q. Minh , and L. S. Vinh . 2018. UFBoot2: Improving the ultrafast bootstrap approximation. Molecular Biology and Evolution 35: 518–522. 10.1093/molbev/msx281 PMC5850222 29077904

[aps370091-bib-0021] Hollingsworth, P. M. , C. D. Preston , and R. J. Gornall . 1998. Euploid and aneuploid evolution in *Potamogeton* (Potamogetonaceae): A factual basis for interpretation. Aquatic Botany 60: 337–358. 10.1016/S0304-3770(97)00101-0

[aps370091-bib-0022] Iida, S. , K. Kosuge , and Y. Kadono . 2004. Molecular phylogeny of Japanese *Potamogeton* species in light of noncoding chloroplast sequences. Aquatic Botany 80: 115–127. 10.1016/j.aquabot.2004.08.005

[aps370091-bib-0023] Ito, Y. , G. L. Robledo , L. Iharlegui , and N. Tanaka . 2016. Phylogeny of *Potamogeton* (Potamogetonaceae) revisited: Implications for hybridization and introgression in Argentina. Bulletin of the National Museum of Nature and Science, Series B 42(4): 131–141.

[aps370091-bib-0024] Jennings, W. B. 2017. On the independent gene trees assumption in phylogenomic studies. Molecular Ecology 26: 4862–4871. 10.1111/mec.14274 28752599

[aps370091-bib-0025] Johnson, M. G. , E. M. Gardner , Y. Liu , R. Medina , B. Goffinet , A. J. Shaw , N. J. C. Zerega , et al. 2016. HybPiper: Extracting coding sequence and introns for phylogenetics from high‐throughput sequencing reads using target enrichment. Applications in Plant Sciences 4: e1600016. 10.3732/apps.1600016 PMC4948903 27437175

[aps370091-bib-0026] Johnson, M. G. , L. Pokorny , S. Dodsworth , L. R. Botigué , R. S. Cowan , A. Devault , W. L. Eiserhardt , et al. 2019. A universal probe set for targeted sequencing of 353 nuclear genes from any flowering plant designed using k‐medoids clustering. Systematic Biology 68(4): 594–606. 10.1093/sysbio/syy086. PMC6568016 30535394

[aps370091-bib-0027] Kalyaanamoorthy, S. , B. Q. Minh , T. K. Wong , A. von Haeseler , and L. S. Jermiin . 2017. ModelFinder: Fast model selection for accurate phylogenetic estimates. Nature Methods 14: 587–589. 10.1038/nmeth.4285 PMC5453245 28481363

[aps370091-bib-0028] Kaplan, Z. , and J. Fehrer . 2006. Comparison of natural and artificial hybridization in *Potamogeton* . Preslia 78: 303–316.

[aps370091-bib-0029] Kaplan, Z. , and J. Fehrer . 2007. Molecular evidence for a natural primary triple hybrid in plants revealed from direct sequencing. Annals of Botany 99: 1213–1222. 10.1093/aob/mcm072 PMC3243585 17478544

[aps370091-bib-0030] Kaplan, Z. , and J. Fehrer . 2009. An orphaned clone of *Potamogeton* × *schreberi* in the Czech Republic. Preslia 81: 387–397.

[aps370091-bib-0031] Kaplan, Z. , and J. Fehrer . 2011. Erroneous identities of *Potamogeton* hybrids corrected by molecular analysis of plants from type clones. TAXON 60: 758–766. 10.1002/tax.603011

[aps370091-bib-0032] Kaplan, Z. , and J. Fehrer . 2013. Molecular identification of hybrids from a former hot spot of *Potamogeton* hybrid diversity. Aquatic Botany 105: 34–40. 10.1016/j.aquabot.2012.11.002

[aps370091-bib-0033] Kaplan, Z. , and J. L. Reveal . 2013. Taxonomic identity and typification of selected names of North American Potamogetonaceae. Brittonia 65: 452–468. 10.1007/s12228-012-9299-0

[aps370091-bib-0034] Kaplan, Z. , and J. Štěpánek . 2003. Genetic variation within and between populations of *Potamogeton pusillus* agg. Plant Systematics and Evolution 239: 95–112. 10.1007/s00606-002-0252-7

[aps370091-bib-0035] Kaplan, Z. , J. Fehrer , and C. B. Hellquist . 2009. New hybrid combinations revealed by molecular analysis: The unknown side of North American pondweed diversity (*Potamogeton*). Systematic Botany 34: 625–642. 10.1600/036364409790139745

[aps370091-bib-0036] Kaplan, Z. , J. Fehrer , and C. Hellquist . 2011. *Potamogeton* ×*jacobsii* (Potamogetonaceae) from New South Wales, Australia – the first *Potamogeton* hybrid from the Southern Hemisphere. Telopea 13: 245–256. 10.7751/telopea20116018

[aps370091-bib-0037] Kaplan, Z. , V. Jarolimova , and J. Fehrer . 2013. Revision of chromosome numbers of Potamogetonaceae: A new basis for taxonomic and evolutionary implications. Preslia 85: 421–482.

[aps370091-bib-0038] Kaplan, Z. , J. Fehrer , V. Bambasová , and C. B. Hellquist . 2018. The endangered Florida pondweed (*Potamogeton floridanus*) is a hybrid: Why we need to understand biodiversity thoroughly. PLoS ONE 13: e0195241. 10.1371/journal.pone.0195241 PMC5880373 29608584

[aps370091-bib-0039] Katoh, K. , and D. M. Standley . 2013. MAFFT multiple sequence alignment software version 7: Improvements in performance and usability. Molecular Biology and Evolution 30: 772–780. 10.1093/molbev/mst010 PMC3603318 23329690

[aps370091-bib-0040] Kent, W. J. 2002. BLAT—The BLAST‐Like Alignment Tool. Genome Research 12: 656–664. 10.1101/gr.229202 PMC187518 11932250

[aps370091-bib-0041] King, R. A. , R. J. Gornall , C. D. Preston , and J. M. Croft . 2001. Molecular confirmation of *Potamogeton* × *bottnicus* (*P. pectinatus* × *P. vaginatus*, Potamogetonaceae) in Britain. Botanical Journal of the Linnean Society 135: 67–70. 10.1111/j.1095-8339.2001.tb02370.x

[aps370091-bib-0042] Kirina, A. D. , P. A. Volkova , M. O. Ivanova , and A. A. Bobrov . 2025. Genetic variability does not support species delimitation within the *Zannichellia palustris* complex in temperate Eurasia. Aquatic Botany 202: e103946. 10.1016/j.aquabot.2025.103946

[aps370091-bib-0043] Larsson, A. 2014. AliView: A fast and lightweight alignment viewer and editor for large data sets. Bioinformatics 30(22): 3276–3278. 10.1093/bioinformatics/btu531 PMC4221126 25095880

[aps370091-bib-0044] Les, D. H. , N. M. Murray , and N. P. Tippery . 2009. Systematics of two imperiled pondweeds (*Potamogeton vaseyi*, *P. gemmiparus*) and taxonomic ramifications for Subsection *Pusilli* (Potamogetonaceae). Systematic Botany 34: 643–651. 10.1600/036364409790139727

[aps370091-bib-0045] Li, W. , and A. Godzik . 2006. CD‐HIT: A fast program for clustering and comparing large sets of protein or nucleotide sequences. Bioinformatics 22: 1658–1659. 10.1093/bioinformatics/btl158 16731699

[aps370091-bib-0046] Lindqvist, C. , J. De Laet , R. R. Haynes , L. Aagesen , B. R. Keener , and V. A. Albert . 2006. Molecular phylogenetics of an aquatic plant lineage, Potamogetonaceae. Cladistics 22: 568–588. 10.1111/j.1096-0031.2006.00124.x 34892900

[aps370091-bib-0047] Mai, U. , and S. Mirarab . 2018. TreeShrink: Fast and accurate detection of outlier long branches in collections of phylogenetic trees. BMC Genomics 19: e272. 10.1186/s12864-018-4620-2 PMC5998883 29745847

[aps370091-bib-0048] McLay, T. G. B. , J. L. Birch , B. F. Gunn , W. Ning , J. A. Tate , L. Nauheimer , E. M. Joyce , et al. 2021. New targets acquired: Improving locus recovery from the Angiosperms353 probe set. Applications in Plant Sciences 9: e11420. 10.1002/aps3.11420 PMC8312740 34336399

[aps370091-bib-0049] Minh, B. Q. , H. A. Schmidt , O. Chernomor , D. Schrempf , M. D. Woodhams , A. Von Haeseler , and R. Lanfear . 2020. IQ‐TREE 2: New models and efficient methods for phylogenetic inference in the genomic era. Molecular Biology and Evolution 37: 1530–1534. 10.1093/molbev/msaa015 PMC7182206 32011700

[aps370091-bib-0050] Murphy, K. , A. Efremov , T. A. Davidson , E. Molina‐Navarro , K. Fidanza , T. C. Crivelari Betiol , P. Chambers , et al. 2019. World distribution, diversity and endemism of aquatic macrophytes. Aquatic Botany 158: e103127. 10.1016/j.aquabot.2019.06.006

[aps370091-bib-0051] Nauheimer, L. , N. Weigner , E. Joyce , D. Crayn , C. Clarke , and K. Nargar . 2021. HybPhaser: A workflow for the detection and phasing of hybrids in target capture data sets. Applications in Plant Sciences 9: e11441. 10.1002/aps3.11441 PMC8312746 34336402

[aps370091-bib-0052] Olsen, J. L. , P. Rouzé , B. Verhelst , Y.‐C. Lin , T. Bayer , J. Collen , E. Dattolo , et al. 2016. The genome of the seagrass *Zostera marina* reveals angiosperm adaptation to the sea. Nature 530: 331–335. 10.1038/nature16548 26814964

[aps370091-bib-0053] Ortiz, E. M. , A. Höwener , G. Shigita , M. Raza , O. Maurin , A. Zuntini , F. Forest , et al. 2026. A novel phylogenomics pipeline reveals extensive topological conflict in the evolution of the angiosperm order Cucurbitales. Systematic Biology syag046. 10.1093/sysbio/syag046 42417730

[aps370091-bib-0054] Petersen, G. , O. Seberg , A. Cuenca , D. W. Stevenson , M. Thadeo , J. I. Davis , S. Graham , and T. G. Ross . 2016. Phylogeny of the Alismatales (Monocotyledons) and the relationship of *Acorus* (Acorales?). Cladistics 32: 141–159. 10.1111/cla.12120 34732019

[aps370091-bib-0055] POWO . 2025. Plants of the World Online. Royal Botanic Gardens, Kew, Richmond, United Kingdom. Website https://powo.science.kew.org/ [retrieved 25 May 2025].

[aps370091-bib-0056] Preston, C. D. , P. M. Hollingsworth , and R. J. Gornall . 1998. *Potamogeton pectinatus* L. × *P. vaginatus* Turcz. (*P.* × *bottnicus* Hagstr.), a newly identified hybrid in the British Isles. Watsonia 22: 69–82.

[aps370091-bib-0068] Ramm, Z., and L. Frost. 2026. Data from: Novel target capture probe set reveals widespread unknown hybridization in North American pondweeds (Potamogetonaceae). Dryad Dataset. 10.5061/dryad.qfttdz0xj [accessed 9 October 2026].

[aps370091-bib-0058] Reznicek, A. A. , and R. S. W. Bobbette . 1976. The taxonomy of *Potamogeton* Subsection *Hybridi* in North America. Rhodora 78: 650–673.

[aps370091-bib-0059] Schmickl, R. , A. Liston , V. Zeisek , K. Oberlander , K. Weitemier , S. C. K. Straub , R. C. Cronn , et al. 2016. Phylogenetic marker development for target enrichment from transcriptome and genome skim data: The pipeline and its application in southern African *Oxalis* (Oxalidaceae). Molecular Ecology Resources 16: 1124–1135. 10.1111/1755-0998.12487 26577756

[aps370091-bib-0061] Steenwyk, J. L. , T. J. Buida , Y. Li , X.‐X. Shen , and A. Rokas . 2020. ClipKIT: A multiple sequence alignment trimming software for accurate phylogenomic inference. PLoS Biology 18: e3001007. 10.1371/journal.pbio.3001007 PMC7735675 33264284

[aps370091-bib-0062] Stevens, P. F. 2001 (and onwards). Angiosperm Phylogeny Website. Version 14, July 2017 [and more or less continuously updated since]. Website http://www.mobot.org/MOBOT/research/APweb/ [accessed 24 September 2026].

[aps370091-bib-0063] Štorchová, H. , R. Hrdličková , J. Chrtek , M. Tetera , D. Fitze , and J. Fehrer . 2000. An improved method of DNA isolation from plants collected in the field and conserved in saturated NaCl/CTAB solution. TAXON 49: 79–84. 10.2307/1223934

[aps370091-bib-0060] Stothard, P. 2000. The sequence manipulation suite: JavaScript programs for analyzing and formatting protein and DNA sequences. Biotechniques 28: 1102–1104. 10.2144/00286ir01 10868275

[aps370091-bib-0064] Wang, Q. D. , T. Zhang , and J. B. Wang . 2007. Phylogenetic relationships and hybrid origin of *Potamogeton* species (Potamogetonaceae) distributed in China: Insights from the nuclear ribosomal internal transcribed spacer sequence (ITS). Plant Systematics and Evolution 267: 65–78. 10.1007/s00606-006-0499-5

[aps370091-bib-0065] Wiegleb, G. , and Z. Kaplan . 1998. An account of the species of *Potamogeton* L. (Potamogetonaceae). Folia Geobotanica 33: 241–316. 10.1007/BF03216205

[aps370091-bib-0066] Wiegleb, G. , H. Brux , and W. Herr . 1991. Human impact on the ecological performance of *Potamogeton* species in northwestern Germany. Vegetatio 97: 161–172. 10.1007/BF00035389

[aps370091-bib-0067] Zhang, C. , and S. Mirarab . 2022. Weighting by gene tree uncertainty improves accuracy of quartet‐based species trees. Molecular Biology and Evolution 39: msac215. 10.1093/molbev/msac215 PMC9750496 36201617

