# Supplementary material for: A novel target capture probe set reveals widespread unknown hybridization in North American pondweeds (Potamogetonaceae)
Source: Appl Plant Sci. 2026 Nov 13:e70091. Online ahead of print. doi: 10.1002/aps3.70091 (PMC13761154; doi:10.1002/aps3.70091)

DOI 10.1002/aps3.70091

Appendix S12: Full topology of concatenated cpDNA genes. Support values at nodes reflect SH-aLRT/ ultrafast bootstrap support.

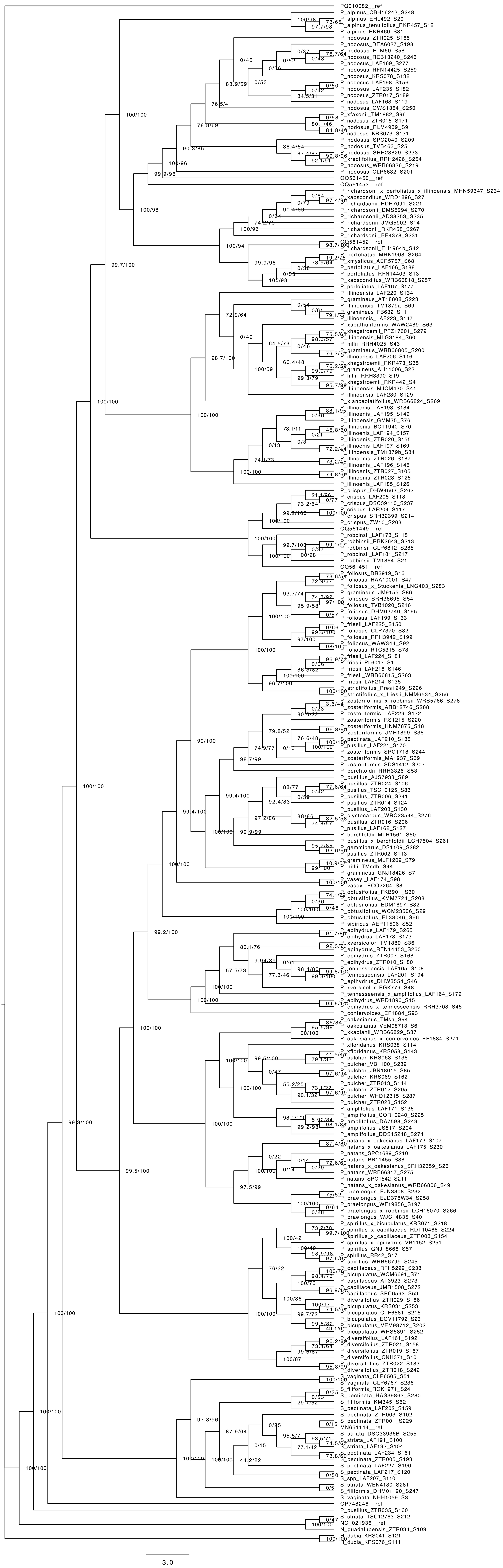

Supplement: Supplementary file 1 [file APS3-9999-e70091-s002.pdf]
