# Supplementary material for: A novel target capture probe set reveals widespread unknown hybridization in North American pondweeds (Potamogetonaceae)
Source: Appl Plant Sci. 2026 Nov 13:e70091. Online ahead of print. doi: 10.1002/aps3.70091 (PMC13761154; doi:10.1002/aps3.70091)

Ramm and Frost—Applications in Plant Sciences 2026 14(6)—Data Supplement S3—Page 1 of 1
DOI 10.1002/aps3.70091

Appendix S13: Full topology for the ITS nuclear ribosomal locus. Support values are omitted for readability.

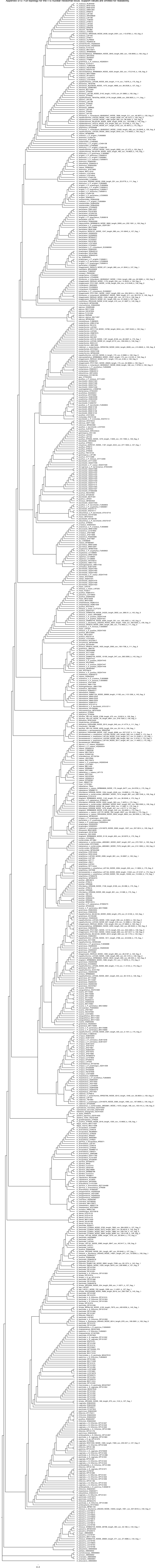

Supplement: Supplementary file 3 [file APS3-9999-e70091-s005.pdf]
